# First Report of a Common Dolphin ( *Delphinus delphis* ) Feeding Alongside Indo‐Pacific Humpback Dolphins ( *Sousa chinensis* ) in the Beibuwan Gulf, China

**DOI:** 10.1002/ece3.71767

**Published:** 2025-07-13

**Authors:** Fuxing Wu, Nan Jin, Minhao Gao, Minding Zhong, Yufei Dai, Yupeng Li

**Affiliations:** ^1^ Laboratory of Marine Biodiversity Third Institute of Oceanography, Ministry of Natural Resources Xiamen People's Republic of China; ^2^ Observation and Research Station of Coastal Wetland Ecosystem in Beibu Gulf Ministry of Natural Resources Beihai People's Republic of China; ^3^ Key Laboratory for Polar Science Polar Research Institute of China, Ministry of Natural Resources Shanghai People's Republic of China

**Keywords:** Beibuwan gulf, common dolphin, feeding, Indo‐Pacific humpback dolphin, mixed group

## Abstract

Research on mixed‐species groups of cetaceans has increased worldwide in recent decades; however, their drivers are not well understood. Although the common dolphin ( *Delphinus delphis* ) and Indo‐Pacific humpback dolphin ( *Sousa chinensis* ) do not form a sympatric community and exhibit different patterns of diet and activity, we observed, for the first time, a mixed‐species group in the Shatian waters of the Beibuwan Gulf, China (a typical habitat for the latter species) on the 3rd and 6th of May 2022. On both occasions, two humpback dolphins were closely accompanied by a common dolphin identified by its distinctive body features. During an approximately 6‐h period of observation, the mixed group exhibited a concentrated use of a very small area in shallow waters and proximity to the coast. Behavior analysis showed that foraging was the most frequently observed activity, followed by socializing, traveling, and resting, which may provide confirmation of the foraging advantage hypothesis for the mixed species group. Our findings are the first to show that the common dolphin and Indo‐Pacific humpback dolphin are mixed‐species groups that expand the known mixed groups of cetaceans.

## Graphical abstract

In this paper, we firstly reported a recent unusual case of a Common dolphin ( *Delphinus delphis* ) mixed group with the estuarine Indo‐Pacific humpback dolphins ( *Sousa chinensis* ) in the Beibuwan Gulf, China. Correspondence behavior analysis showed that foraging was the most frequently observed activity, which might confirm the foraging advantage hypothesis for the mixed‐species group. To our knowledge, our findings expanded the known mixed groups of cetaceans and provided an important scientific basis for the behavior of these two species.

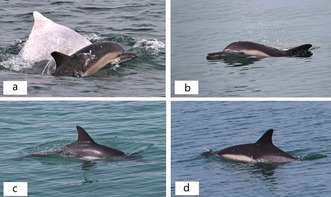

## 1 Introduction

Interspecific interactions and mixed‐species groups have been reported in many zoological taxa, such as fish, birds, and mammals (Heymann and Hsia 2015; Kiffner et al. 2014; Veit and Harrison 2017; Zou et al. 2018). Mixed‐species events have also been postulated to be a common phenomenon in the wild in cetaceans, with over 33 species of cetaceans and over 216 different species mixed‐group events being recorded around the world (Syme et al. 2021). However, most events were observed in all the major ocean basins, except for the Southern Ocean, with most records over the continental shelf around oceanic islands in the open ocean; however, the species in the shallow coastal or estuarine waters have rarely been observed (Koper and Plön 2016; Syme et al. 2021).

The main functional explanations for cetacean mixed‐species group formation are the anti‐predator, foraging, and social advantage hypotheses (Stensland et al. 2003; Syme et al. 2021). Therefore, mixed‐species events in the wild in cetaceans normally associate more frequently with other species with similar foraging niches, whereas those occupying a different vertical foraging niche and preferred prey species may be associated with similar species, especially for *Delphinidae* species (Curcio et al. 2023; Syme et al. 2021).

The common dolphin ( *Delphinus delphis* ) is one of the most abundant species of small cetaceans, mainly inhabiting the tropical and warm‐temperate waters of the Pacific, Atlantic, and Indian Oceans, as well as most seas, and occupies a wide range of habitats, from nearshore areas to the open ocean (Braulik et al. 2021; Yoo et al. 2023). Although widely distributed, they may be dietary specialists, feeding on schooling fish from Clupeidae, Myctophidae, Centracanthidae, and Sparidae (Milani et al. 2021; Pusineri et al. 2007). The common dolphin has often been observed to form large schools of several hundred to thousands of individuals (Saintignan et al. 2020), but small groups rarely exceeding 20–35 animals are characteristic of coastal communities (Milani et al. 2021; Petroselli 2006). A seasonal change in the distribution and density of the common dolphin was observed because of a change in feeding strategies at different times of the year such that groups followed the migration movement of food (Milani et al. 2021).

The common dolphin is one of the most commonly reported species in mixed‐species groups in previous studies (Syme et al. 2021) and mixed groups between common dolphins and several other cetacean species that overlap ecological niches are well documented (Syme et al. 2021). Associations with schools of pilot whales (*Globicephala* spp.), striped dolphins ( *Stenella coeruleoalba* ), dusky dolphins ( *Lagenorhynchus obscurus* ) and several other small delphinids have been observed (Bonizzoni et al. 2019; Curcio et al. 2023; Frantzis and Herzing 2002; Syme et al. 2021), but all of which did not include shallow‐water inshore or estuarine species.

Compared with the common dolphin, the Indo‐Pacific humpback dolphin ( *Sousa chinensis* ) is a coastal dolphin species and is described as a resident, widely found in the estuarine and inshore areas of the western Indian to western Pacific Ocean, generally inhabiting shallow and muddy waters where the distance is generally within 7 km of shore and water depth seldom exceeds 15 m (Jefferson and Rosenbaum 2014; Karczmarski et al. 2000; Lin et al. 2022). It is listed as “Vulnerable” in the International Union for Conservation of Nature (IUCN Red List) and has been listed as a Grade I National Key Protected Animal by the Chinese State Council since 1988 due to much of its habitat being subject to intensive anthropogenic disturbance and because of a continued decline in its population during recent decades in different subregions, becoming rare or locally absent (Jefferson and Smith 2016; Wu et al. 2014).

Even though populations of Indo‐Pacific humpback dolphins are already well known and have been subject to long‐term studies (Jefferson and Smith 2016; Jutapruet et al. 2015; Lin et al. 2022), mixed‐species events in the wild between this species and other cetacean species have rarely been seen. The Indo‐Pacific finless porpoises ( *Neophocaena phocaenoides* ) are the only reported cetacean species living in sympatry, with overlapping ranges and interspecies affiliations with the Indo‐Pacific humpback dolphin (Meng et al. 2022; Xu et al. 2012), with one reported case of Indo‐Pacific humpback dolphins assisting a finless porpoise calf in breathing off Xiamen, China (Wang et al. 2013).

Although both Indo‐Pacific humpback dolphins and common dolphins have been well studied worldwide, the two species have never been observed mixed together (Syme et al. 2021). Therefore, it was unusual for these two allopatric species to form mixed groups. However, we report the first sighting of the common dolphin distributed in estuarine waters and the first case of an individual mixed group with Indo‐Pacific humpback dolphins, and followed them for several days in Shatian Waters, Beibuwan Gulf, China.

## 2 Materials and Methods

**FIGURE 1:**
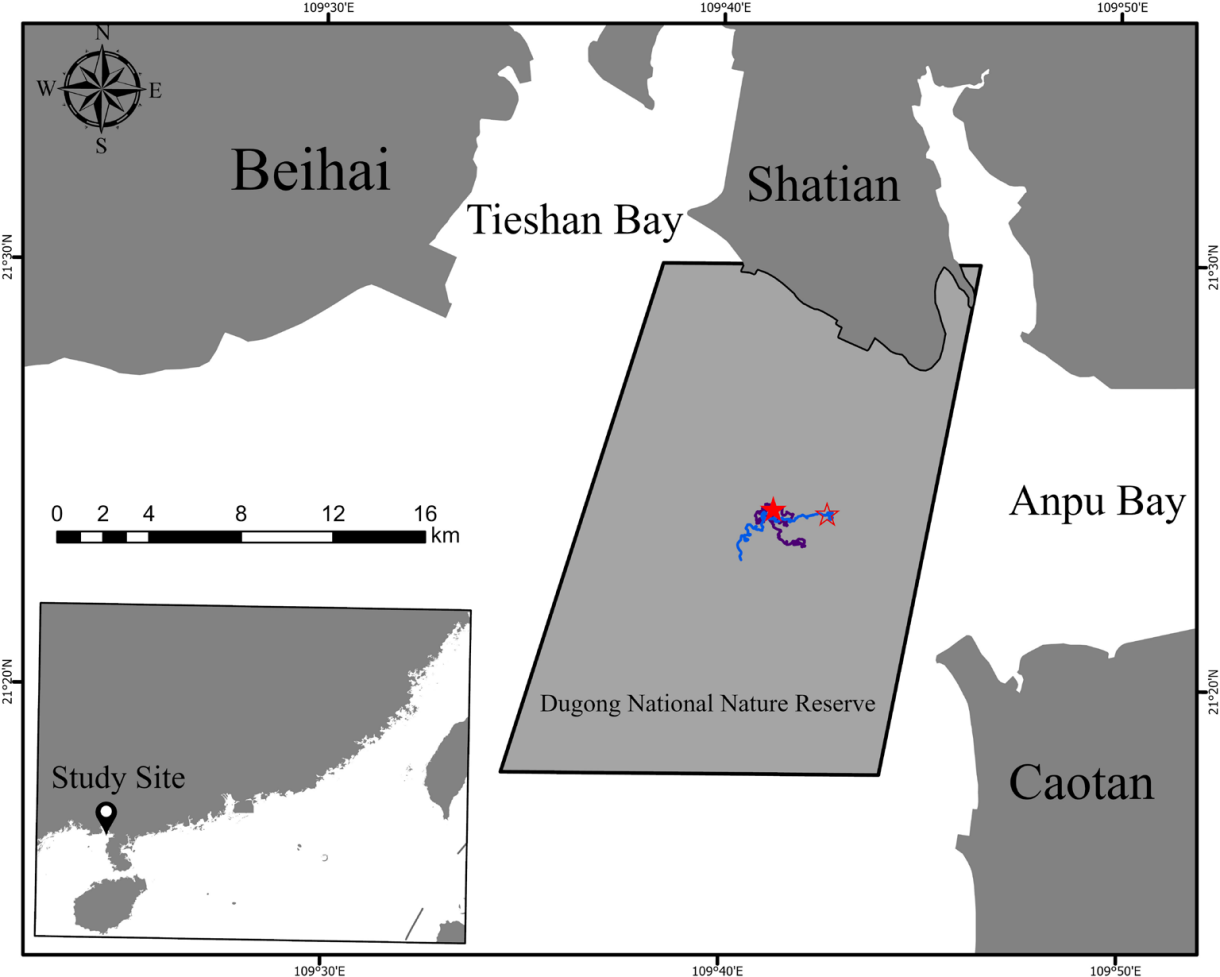
Locations of the common dolphin ( *Delphinus delphis* ) and Indo‐Pacific humpback dolphin ( *Sousa chinensis* ) in mixed‐species groups at Shatian waters of Beibuwan Gulf, China. The red solid pentagram shows the location of the common dolphins found on 3 May 2025 and the purple line is the tracking line. The hollow pentagram shows the location of the common dolphins found on another day and the blue line is the tracking line.

A total of 41 boat‐based line field surveys of marine mammals were conducted onboard an 11‐m‐long wooden boat powered by inboard engines at seasonal intervals between 2020 and 2022 in the waters of Hepu Dugong National Nature Reserve in the Beibu Gulf, China (Figure 1, which was produced in the ArcGIS Pro 3.0.2), which represents one of the crucial distribution areas of Indo‐Pacific humpback dolphins and Indo‐Pacific finless porpoises with no verified field observations of Dugong (*Dugong*) after 2000 (Chen et al. 2016; Meng et al. 2022; Xu et al. 2012), to investigate the distribution and abundance of these two species. Seasonally (every 3 months) a survey was conducted for approximately 4–5 days in a row, and the boat was at a survey speed of approximately 10–13 km/h under good weather conditions (Beaufort scale ≤ 3). The position of the boat was continually recorded at 1‐s intervals using a handheld Garmin GPS 76SCx GPS receiver (Garmin Ltd., USA). Whenever a group of dolphins was sighted, the speed of the boat was reduced, and the engine was stopped once an appropriate distance was reached, at which point photographs were taken (Serres et al. 2023). Photographs of the dorsal fins and bodies of each dolphin were taken using Canon EOS 1DX III digital cameras (Canon, Tokyo, Japan) fitted with 100–400 mm lenses. Individual dolphins were identified based primarily on the pattern of spots, nicks, notches, and pigments on and/or around dorsal fins (Wu et al. 2014; Wu et al. 2025). All photographs of the identified individuals were saved in the Shatian‐Caotan Indo‐Pacific humpback dolphin photo‐ID database managed by the Third Institute of Oceanography, Ministry of Natural Resources. Primary data, including the name of the observer, date and time, GPS coordinates of the research vessel, species, group size, and behavior, were recorded.

Due to the small and stable group and all dolphins being identifiable, behavioral patterns were recorded every 5 min (defined as one behavioral state encounter) using the focal follow sampling criteria when dolphins were encountered (Mann 1999). Encounters with dolphins involved an unmanned aerial vehicle (UAV: DJI Mavic 3) for morphometric measurements and behavior recordings (Serres et al. 2023). The lens of the unmanned aerial vehicle was pointed vertically down to ensure that the Indo‐Pacific humpback and common dolphin were in a horizontal position in the pictures when they were swimming in a synchronized manner. Behavioral states were recorded in broad categories such as feeding/foraging, traveling, socializing, and resting/milling (Table 1) following definitions based on the existing literature (Serres et al. 2023). Furthermore, aerial behavioral events, including breaches, side breaches, tail slaps, spy hops, and head slaps, as described by Serres et al. (2023) and Curcio et al. (2023) were also recorded.

**TABLE 1:** Description of dolphin surface behavioral categories described in this article.

Behavioral categories	Description
Feeding/foraging	To describe any effort to capture and consume prey including repetitive prolonged dives in one location; chasing fish; making circles; having a parallel swimming formation, with fast, directional, and synchronized movements; fish leaping out of the water; and even dolphins with fish in their mouths
Traveling	Directional and persistent movement at speed comprising either slow or fast
Socializing	Inter‐individual interactions within a group, animals engaging in body contact, with frequent vigorous movements and aerial behaviors such as breaching. This also included sexual and aggressive behaviors
Resting/milling	Animals exhibit inactive non‐directional slow movements within the same location or staying still at the surface

Oceanographic variables were measured when the dolphins were sighted. Water depth (m) for each dolphin sighting was recorded by placing a SpeedTech portable sounder on the water surface (Laylin Associates Ltd. USA) (Wu et al. 2025). Sea surface temperature (°C), dissolved oxygen (mg/L), surface salinity (practical salinity units, PSU), pH, and turbidity (Nephelometric Turbidity Units, NTU) were also measured by placing a YSI EXO‐S multiparameter water quality sonde in the surface of the seawater (Xylem Inc., USA) (Wu et al. 2025). The distance of each dolphin‐sighting location point where the dolphins had been initially sighted to the shore was calculated using a proximity analysis tool module in ArcMap 10.2. The means of the oceanographic variables were presented as mean ± standard error (SE). The calculations were conducted in R 4.0.4.

## 3 Results

**FIGURE 2:**
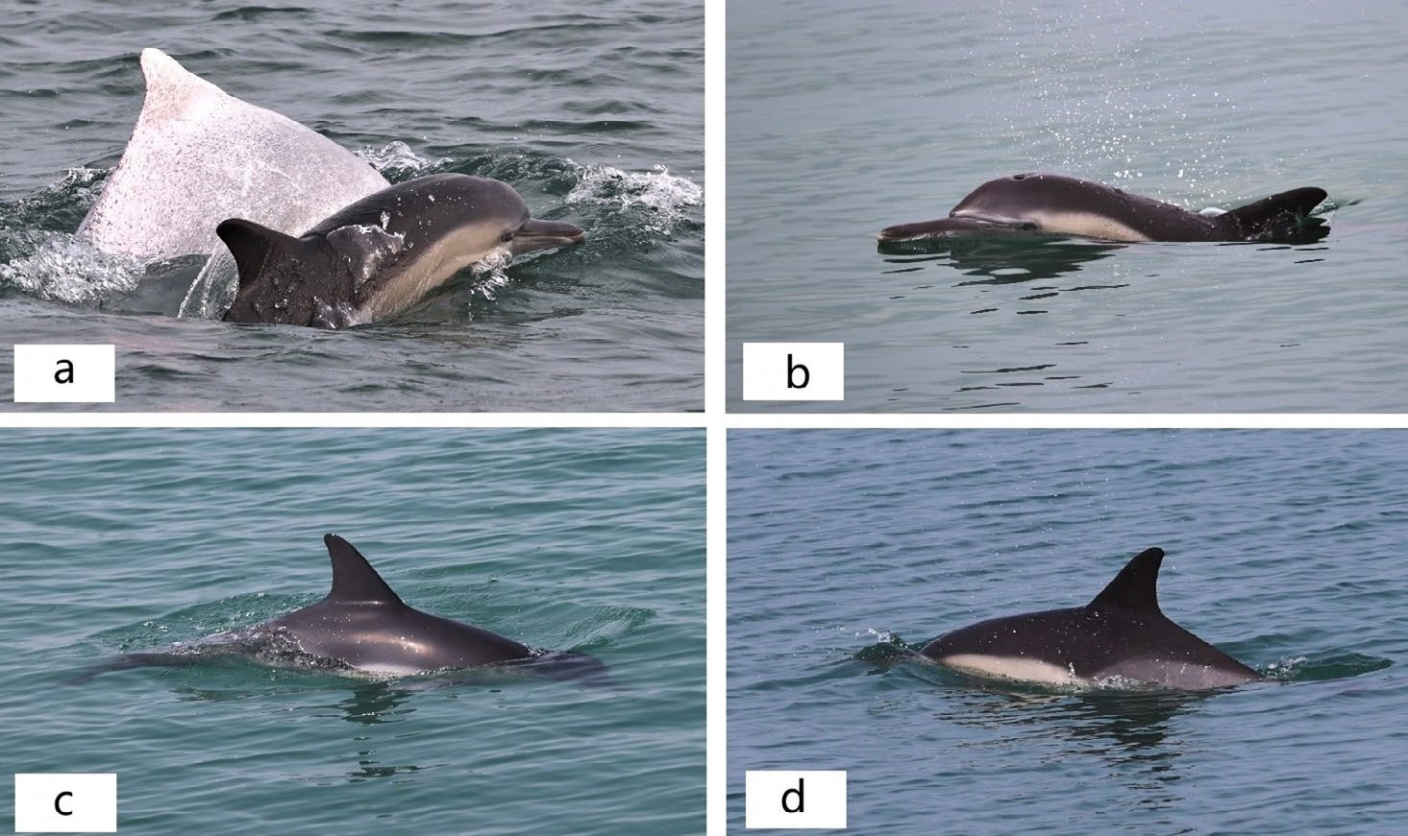
Body features of the common dolphin ( *Delphinus delphis* ) found in the Shatian waters of Beibuwan Gulf. (a) The common dolphin was following the Indo‐Pacific humpback dolphin; (b) There was a deep crease between the melon and beak of the common dolphin; (c) The dorsal fin of the common dolphin was tall and moderately falcate; (d) There was an hourglass pattern on the side of the common dolphin, forming a V below the dorsal fin.

On May 3, 2022, during a boat‐based survey of cetaceans in the Shatian‐Caotan waters (Beibuwan Gulf, China), a dark gray dolphin following a group of two Indo‐Pacific humpback dolphins was sighted (Figure 2a). According to observations and photographs, the body shape of the dolphin was slender, and there was a deep crease between the melon and beak (Figure 2b). The dorsal fin of the individual was tall and moderately falcate (Figure 2c) and was characterized by an hourglass pattern on the side, forming a V below the dorsal fin (Figure 2d). Therefore, the dolphin encountered in the present study was identified as a common dolphin, based on these body features (Jefferson et al. 2015).

In total, two spotted adult Indo‐Pacific humpback dolphins were identified in the group based on both on‐site observations and subsequent photographs and video analysis. When comparing photographs with the Shatian‐Caotan Indo‐Pacific humpback dolphin photo‐ID database preserved at the Third Institute of Oceanography, Ministry of Natural Resources, the two individuals were identified and sighted during the field surveys in 2020–2022; they were encoded as SC0001 and SC0019, respectively, with SC0001 being resighted nine times and SC0019 being resighted six times. Referring to the adult Indo‐Pacific humpback dolphin, which reaches a length of approximately 230–269 cm long in Chinese waters (Huang et al. 1997; Zhai et al. 2010), the body length of the common dolphin was estimated to be 134–151 cm relative to two adult humpback dolphin pictures taken by a drone. Therefore, the common dolphin was judged as a subadult because it was over 164 cm in size (Jefferson et al. 2015). There were no obvious scars, notches, or teeth marks on the body of the common dolphin, but there were temporary marks, such as small and white patches, on the right side of the caudal peduncle and the bottom of the dorsal fin.

The original GPS location of the first sighting of the mixed‐species groups was 21°24′14.4″N, 109°41′18.7″E, with the distance between the sighting location point and shoreline being about 9.01 km. They used a very small area in shallow waters in proximity to the coast after tracking for more than 3 h, from 1417 to 1730 h (UTC + 08:00), with the tracking line being approximately 15 km (Figure 1). Observations were stopped when the sea state changed to Beaufort 4–5 and sight of the dolphins was lost. Three days later, the mixed‐species group was encountered again according to photograph IDs obtained during surveys in waters closer to the coastline, with the distance between the sighting location point and the shoreline being approximately 7.63 km. Behavioral observations of the mixed‐species group lasted for approximately 3 h. At the locations where the mixed‐species group was found, the water depth was 8.5 ± 1.22 m, the sea surface temperature was 27.4°C ± 0.8°C, the dissolved oxygen was 6.67 ± 0.22 mg/L, the salinity was 32.03 ± 0.07 psu, the pH value was 8.08 ± 0.02, and the turbidity degree was 1.9 ± 1.23 NTU.

**FIGURE 3:**
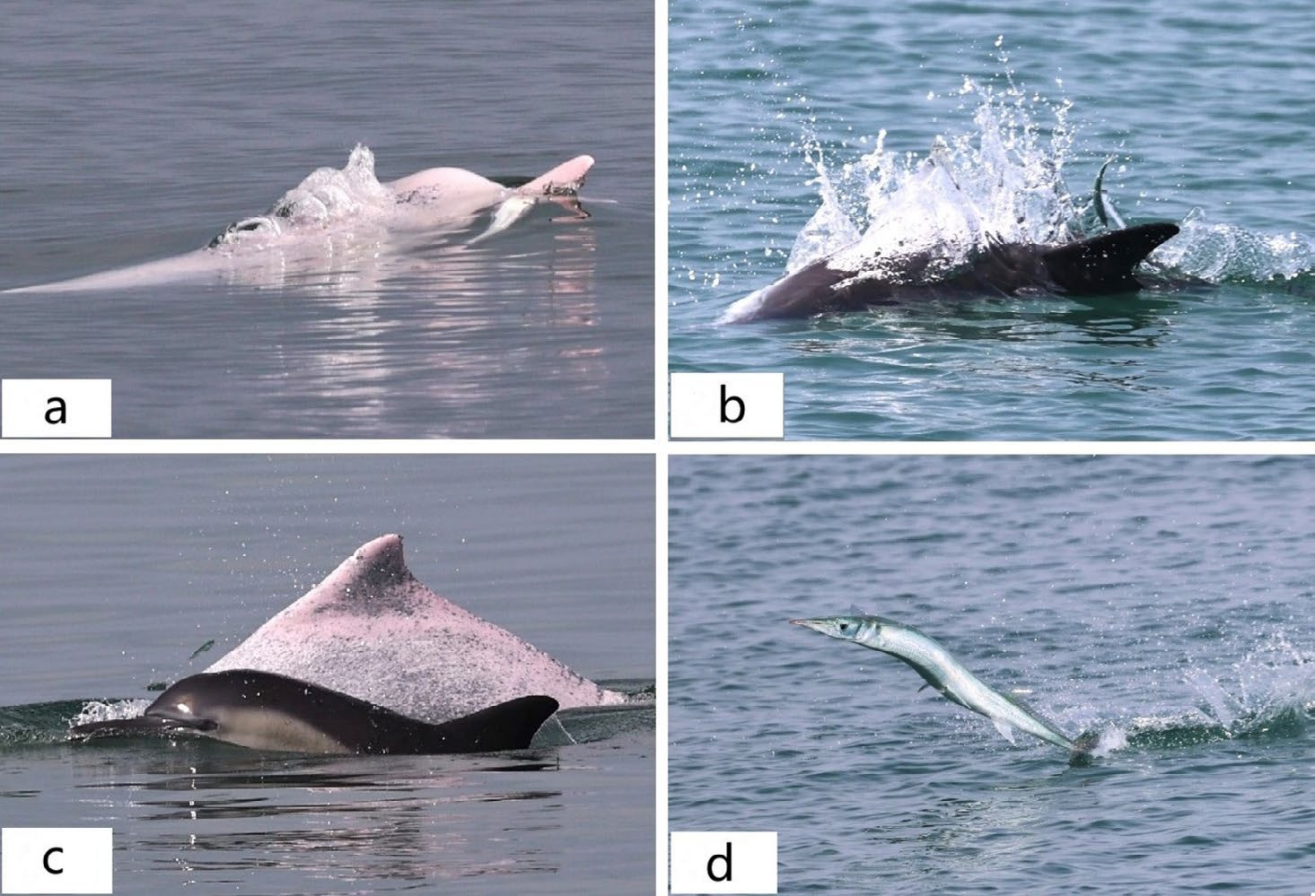
The common dolphin ( *Delphinus delphis* ) and Indo‐Pacific humpback dolphin ( *Sousa chinensis* ) displayed the surfacing behavior of chasing fish or consuming prey. (a) The Indo‐Pacific humpback dolphin was consuming prey; (b) The common dolphin was chasing fish; (c) Both the Indo‐Pacific humpback dolphin and common dolphin were chasing fish; (d) The fish was being chased by dolphins.

Five behavioral state encounters were excluded from the analysis because the team was unable to follow the dolphin and lacked behavioral observation data, resulting in 61 analyzed behavioral state encounters. The most frequently observed behavioral states were 43 encounters of foraging activity (70.49%), with both species displaying the surfacing behavior of chasing fish or consuming prey (Figure 3), followed by nine encounters of socializing (14.75%), with both species engaging in body contact or found very closely. Traveling (13.12%) was the third most frequently observed behavioral state, with eight encounters and both species traveling in the same direction, while resting (1.64%) was rare, with only one instance of this behavior being recorded. The encounter number of foraging activity was significantly higher than those of socializing or traveling (chi‐square test, *p* value < 0.01). In addition, a variety of aerial behaviors of these two species were observed, with the common dolphin displaying four breaches and one head slap, and the Indo‐Pacific humpback dolphin displaying only three tail slaps.

## 4 Discussion

Numerous delphinid species occurring in overlapping ecological niches that form mixed‐species groups are well documented, with the common dolphin being one of the most commonly reported species that forms mixed groups with other cetacean species (Syme et al. 2021). Previous studies have indicated that the common dolphin is associated with at least 27 different species globally, none of which are coastal or estuarine species (Syme et al. 2021). To the best of our knowledge, the present report is globally the first report of a common dolphin occurring in an estuarine area that forms a mixed group with the resident Indo‐Pacific humpback dolphin.

Studies on the functional explanations of cetacean mixed‐species group formation are still limited and there is not one single explanation for why mixed‐species groups are formed (Syme et al. 2021). Only 13.3% of the 203 studies on cetacean interactions discussed potential functional explanations based on specific observations or investigations, and several hypotheses have been proposed (Syme et al. 2021), including improving foraging (Koper and Plön 2016; Quérouil et al. 2008), gaining social benefits (Frantzis and Herzing 2002; Syme et al. 2021), and reducing predation risk (Kiszka et al. 2011). However, cetacean interactions are complex and diverse, and many functions and drivers may play different roles in different contexts. Of these hypotheses, the most widely supported is that interactions between cetaceans allow the animals to increase foraging success (Quérouil et al. 2008), which might also be supported in our study as the most frequently observed behavioral states were foraging activity, and many surfacing behaviors of chasing fish or consuming prey were observed.

However, dietary divergence and feeding habits vary greatly between common and Indo‐Pacific humpback dolphins. As would be expected for a species occupying a wide range of habitats and having high physiological tolerance, the stomach contents of common dolphins show that this species is an epipelagic predator that opportunistically feeds on a wide variety of prey, varying from pelagic prey to neritic prey (de Pierrepont et al. 2005; Milani et al. 2021; Pusineri et al. 2007). In comparison, the Indo‐Pacific humpback dolphin mainly feeds on benthic fishes, usually at water depths of less than 15 m (Barros et al. 2004; Huang et al. 1978). Indo‐Pacific humpback dolphins adapt to shallow water, and perhaps the common dolphin's adapting behaviorally to the humpback dolphin may help to locate its prey and increase the accessibility of prey. Feeding in groups may greatly increase predation efficiency and decrease energy expenditure, as suggested in a previous study (Stensland et al. 2003).

Considering that common dolphins are often regarded as highly mobile animals (Mirimin et al. 2009), and have more surface behavioral events, exhibiting a range of coordinated feeding strategies, with the most frequently reported feeding techniques of this species being the carouseling of shoaling fish (Neumann and Orams 2003; Stockin 2008), sharing shallow water habitats with common dolphins and adopting their foraging behavior may also benefit Indo‐Pacific humpback dolphins through herding food resources. As observed in our study, common dolphins act closer to the water surface, while Indo‐Pacific humpback dolphins feed on or close to the seabed in small groups, with foraging behavior indicated by repeated dives in varying directions in a particular location, as observed in other studies (Bearzi et al. 1999; Spitz et al. 2012).

Historic whaling activities and sporadic stranding or incidental catch records indicated that the common dolphin could be found in all the sea waters of China and was mainly found in the East China Sea and South China Sea (Wang 2012; Wang et al. 2003). However, observations of common dolphins in Chinese waters are scarce, resulting in little information available about the distribution and critical habitats of the species throughout the entire area of Chinese waters, which results in a lack of basic knowledge of their status and biology.

In China, the common dolphin has been listed as a key second‐level nationally protected animal since 1988. Although several incidental capture events by fishers have been recorded, there have been no estimates of the impact fisheries might have on this species (Wang 2012; Wang et al. 2003). Therefore, in addition to collaboration with fishermen to spread awareness and implementation of the ecosystem and local ecological knowledge approach (Wu et al. 2014), it is recommended that additional research on the distribution, abundance, population structure, ecology, and conservation problems caused by various anthropogenic activities is needed to better understand and protect this species in Chinese waters in future research.

## Author Contributions

**Fuxing Wu:** conceptualization (lead), data curation (lead), formal analysis (lead), funding acquisition (lead), investigation (lead), methodology (lead), project administration (lead), resources (lead), software (lead), supervision (lead), validation (lead), visualization (lead), writing – original draft (lead), writing – review and editing (equal). **Nan Jin:** investigation (equal), writing – review and editing (equal). **Minhao Gao:** investigation (equal), writing – review and editing (equal). **Minding Zhong:** investigation (equal), writing – review and editing (equal). **Yufei Dai:** investigation (equal), writing – review and editing (equal). **Yupeng Li:** writing – review and editing (equal).

## Conflicts of Interest

The authors declare no conflicts of interest.

## Data Availability

The original GPS location of the first sighting of the mixed‐species groups of the common dolphin (*Delphinus delphis*) and Indo‐Pacific humpback dolphins (*Sousa chinensis*) has been inserted in the manuscript.
